# Introduction to “Immunotherapies for Multiple Myeloma”

**DOI:** 10.3390/ph13110396

**Published:** 2020-11-17

**Authors:** Massimo Offidani, Maria Teresa Petrucci

**Affiliations:** 1 Clinica di Ematologia, Azienda Ospedaliero-Universitaria, Ospedali Riuniti di Ancona, via Conca, 71, 60126 Ancona, Italy; 2 Ematologia, Dipartimento di Medicina Traslazionale e di Precisione, Azienda Ospedaliera Policlinico Umberto I, Università “Sapienza” di Roma, via Benevento 6, 00161 Roma, Italy; petrucci@bce.uniroma1.it

Multiple myeloma (MM) is the second most common hematological cancer after diffuse large B-cell lymphoma, accounting for about 10% of all blood cancers [1]. Since 1990, the global incidence has increased by 126%, and it typically occurs in the elderly, with 85 and 60% of diagnoses made in individuals over 55 and 65 years of age, respectively [2]. 

MM is a hematologic disease characterized by a clonal proliferation of aberrant plasma cells in the bone marrow, associated with excessive production of monoclonal immunoglobulins in blood and urine. This monoclonal protein (M-protein) is able to induce various types of impaired immune function, and in addition, MM typically causes end-organ damage consisting of specific signs and symptoms, such as hypercalcemia, renal insufficiency, anemia, and lytic bone lesions (CRAB criteria) [3]. 

The treatment options in MM has changed over the last two decades, and the therapy armamentarium of effective anti-myeloma drugs, used both at diagnosis and at relapse, has expanded significantly with the introduction of several novel agents, including proteasome inhibitors (PIs, bortezomib, carfilzomib, and ixazomib), immunomodulatory drugs (IMiDs, thalidomide, lenalidomide, and pomalidomide), and histone deacetylase blocked (HDAC, panobinostat) [3,4]. With improved treatment regimens and the use of high-dose chemotherapy, followed by autologous hematopoietic stem-cell transplantation (ASCT), median overall survival (OS) of newly diagnosed MM (NDMM) patients eligible for ASCT is 6–8 years. At present, approximately 50% of MM patients are still alive 5 years after the initial diagnosis, even though the OS of these patients, both young and elderly, has steadily risen over the last few years. One-third of the patients can live more than 10 years, according to disease risk staging (International Staging System—ISS) and cytogenetic abnormalities (Revised International Staging System—R-ISS) that have an impact on prognosis [5]. However, despite the availability of these new drugs and high-dose chemotherapy followed by ASCT, MM still remains largely an incurable disease, even in standard risk patients. Despite most of the patients achieving response with the initial treatment, the majority of them experience a relapse [3]. Natural history of MM is characterized by an alternation of phases of disease remission, followed by phases of relapse. The duration of remission phases tends to progressively decrease at every subsequent relapse, until MM becomes refractory to all available drugs. 

In newly diagnosed eligible MM patients, a large proportion of patients with MM can now achieve a hematologic complete response (CR), thanks to recent treatment advances that include the combined use of immunomodulatory drugs and proteasome inhibitors, in association with high-dose chemotherapy with ASCT. Unfortunately, despite this, most MM patients achieving CR eventually relapse, suggesting that a very small, but clinically relevant, proportion of clonal plasma cells persist in the bone marrow after therapy, and these cells are not detected by current techniques. Over the last few years, newly specific assays with greater sensitivity have been developed to detect minimal residual disease (MRD), including multiparameter flow cytometry (MFC), allele-specific oligonucleotide quantitative polymerase chain reaction (ASO-qPCR), and next-generation sequencing (NGS) techniques [6]. 

Currently, the treatment goal of first-line therapy is the achievement of MRD negativity. A large-cohort analysis confirms that MRD status is a value surrogate marker both for progression-free survival (PFS) and OS in patients with MM, including those who had achieved a CR. About 50–80% of transplants-eligible and 15–30% of transplant-ineligible patients achieve MRD negativity after first line treatment based on the new drugs combination. In addition, all recent studies confirmed that MRD has an impact on MM patient’s outcome, regardless the treatment used [3,6].

In order to prevent and to overcome the natural relapse or treatment resistance, ongoing challenges to identify novel therapeutic strategies and new compounds with different mechanisms of actions are necessary. Over the last few years, scientific attention has been drawn increasingly to immunotherapeutic strategies with the potential for improved targeting for MM clonal plasma cells to maintain a prolonged response, and to achieve a possible cure for MM patients. The field of immune therapy has been accelerating in the treatment of hematological disease and also has a central role in MM. 

Allogenic stem-cell transplantation (allo-SCT) can be considered the first immunotherapy offering the potential for prolonged survival time in MM patients. T cells of the donor, co-administered with the stem cells, can recognize minor histocompatibility antigen-presenting myeloma cells in the human leukocyte antigen (HLA)-matched setting and are able to eliminate clonal plasma cells, resulting in the so-called graft-versus-myeloma effect [7]. Long-term survival in a small subset of MM patients after allo-SCT demonstrates the potential role of immunotherapy in MM, but allo-SCT is also associated with a high rate of treatment-related mortality [8], and it should be purposed within clinical trials.

Immunotherapy, either passive, as in the case of monoclonal antibodies (MoAbs) and cellular products directed against clonal plasma cells, or active (when a patient’s immune system is stimulated to induce an immune response against tumor cells) is currently the key strategy for the treatment of hematologic malignancies, especially MM [3]. 

MoAbs, in particular, have entered clinical practice for the treatment of MM. Clonal plasma cells show several surface molecules that have been explored as potential targets for MoAbs. These include CD38, CD40, CD138, CD56, CD54, IL-6, PD1, CD74, CD162, b2-macroglobulin, kappa light chain, B-cell maturation antigen (BCMA), ganglioside GM-2, and the signaling lymphocyte activation molecule F7 (SLAMF7). Ideally, these MoAb-therapeutic targets should be predominantly expressed on aberrant plasma cells and not on normal hematopoietic cells or non-hematopoietic tissue [9]. In addition, MoAbs may modulate the bone marrow microenvironment, resulting in the enhancement of the host antitumor immune response. MoAb therapies involve several mechanisms of action, including direct cytotoxic effects on the clonal plasma cell, antibody-dependent cellular cytotoxicity (ADCC), antibody-dependent cell-mediated phagocytosis (ADCP) and complement-dependent cytotoxicity (CDC).

In MM, these immunotherapeutic agents can be used as monotherapy, or, even more successfully, in combination with other anti-MM drugs to further improve depth and duration of response by preventing the outgrowth of resistant clones. Two antibody targets, SLAMF7 and CD38, are of particular interest in MM. The first in class Elotuzumab, a humanized immunoglobulin IgG_1_ monoclonal antibody that targets SLAMF7 in combination with lenalidomide and dexamethasone (Rd) was approved by the Food and Drug Administration (FDA) in 2015 and by the European Medical Agency (EMA) in 2016 for the treatment of relapse-refractory MM (RRMM) [10]. More recently, Elotuzumab has been combined with pomalidomide and dexamethasone and with bortezomib and dexamethasone in triplet therapies, both resulting in superior PFS compared with duplets standard Pd and Vd [11,12].

The second important antibody target in MM is CD38 and several CD38-targeting antibodies have been developed. Daratumumab, the first humanized IgG_1_K monoclonal antibody targeting CD38, revealed anti-MM efficacy as monotherapy, as well as in combination with Rd and VD (bortezomib and dexamethasone) in heavily pretreated RRMM patients, which resulted in FDA and EMA approval [8]. Daratumumab was evaluated with pomalidomide and dexamethasone (Dara-Pd) and, in a preliminary phase II trial, this combination showed a better response in heavily pretreated patients, compared to Pd. Thus, following the encouraging results of this study, the triplet Dara-Pd received accelerated approval by FDA in 2017 for RRMM patients who previously received both an IMiD and a PI [13]. Definitive results will come from the phase III trial APOLLO (NCT03180736) comparing Dara-Pd versus Pd in RRMM patients. Based on the results of the randomized phase III trial comparing D-Rd vs. Rd, the FDA approved daratumumab in combination with lenalidomide and dexamethasone in newly diagnosed MM not eligible for ASCT [14]. In addition, Dara-VMP has recently been approved by both the FDA and EMA, thus becoming one of the standards of care for transplant-ineligible patients with newly diagnosed MM [15].

Daratumumab has also been incorporated in the induction, consolidation, and maintenance approach, in association with standard of care treatment based on VTd, (bortezomib, thalidomide, and dexamethasone) as a frontline therapy for NDMM transplant-eligible patients. In September 2019, based on the encouraging results of the CASSIOPEIA trail, the FDA approved frontline Dara-VTd as induction for transplant-eligible patients [16]. Other ongoing phase II/III trials are evaluating the front-line daratumumab in transplant-eligible patients, in combination with VRd (EMN17/PERSEUS/GRIFFIN trials) and VCd (EMN18 trial) as induction, consolidation, and maintenance. 

Similarly, to daratumumab, isatuximab, another IgG-k chimeric monoclonal antibody targeting CD38, showed a promising efficacy when administered as a single agent or in association with other specific drug in heavily pre-treated MM patients [17]. A phase III study (ICARIA) comparing isatuximab plus pomalidomide and dexamethasone versus pomalidomide and dexamethasone showing a consistent benefit in terms of ORR and PFS for the triplet arm compared to the control group [18]. Other MoAbs, with different targets, are under investigation in MM treatment, for example, siltuximab (anti-interleukin 6) and inhibitors of programmed cell death protein 1 (PD-1)/programmed cell death ligand 1 (PD-L1). PD-1/PD-L1 pathway is a negative regulator of immune activation, and recently, it was demonstrated that the combination of lenalidomide plus PD-1/PD-L1 inhibitors is associated with increased apoptosis of MM clonal plasma cells. However, there are few limited data from clinical trials of PD-1/PD-L1 MoAbs in MM patients [19]. The FDA discontinued phase III trials (KEYNOTE-183) that compared Pd with or without pembrolizumab in RRMM patients due to increased risk of death [20]. 

Summing up, the field of immune therapy has also taken center stage in MM and some immunotherapies are currently approved for the treatment of MM, while several others immunotherapeutic approaches are under investigations and represent a very interesting area of experimental trials. Clinical trials using bispecific antigen-directed CD3 T-cell engager (BiTE), antibody–drug conjugate (ADC), and chimeric antigen receptor T-cell (CAR-T) therapy are ongoing. 

Specifically, ADCs are monoclonal antibodies bound by a chemical linker to specific cytotoxic compound direct against surface antigens of the target cells. ADCs target cells expressing their specific antigen and are internalized releasing the cytotoxic compound leading to cell death. In MM, the safety, tolerability, and preliminary clinical results of B-cell maturation antigen (BCMA)–ADC, a novel antibody toward specific plasma cells antigen (BCMA) conjugates to the microtubule-disrupting agent monomethyl auristatin F (Belantamab Mafodotin), were analyzed in phase I, II, and III trials, showing remarkable activity in patients with advanced MM [21,22,23,24], receiving FDA and EMA approval in this setting in 2020. Other ADCs for MM patients are under investigation in ongoing trials.

Instead, bispecific monoclonal antibodies are engineered compounds that are able to redirect T and NK cells toward tumor cells, in order to restore the immune suppressor activity of the natural immune system against pathological cells. BiTe links the invariant part of CD3 of the T cell receptor on T cells and specific surface antigen on pathological cells leading to T cell proliferation and activation and tumor cells apoptosis. Among several potential target, expressed on clonal plasma cells, BCMA represents the most important and encouraging target. AMG 420, the first anti-BCMA BiTe, binds to CD3+ T cells and BCMA+ MM cells, which make a cross linking between both cells to induce MM cells apoptosis. AMG 420 is under investigation in phase I trials in RRMM patients, and it has shown high efficacy by depleting BCMA+ MM clonal plasma cells [25]. Now, a similar compound with more convenient administration, AMG-701, and many others such as BiTe are under investigation [26,27].

CAR-T therapy is a very exciting treatment for RRMM. CARs are fusion proteins incorporating an antigen-recognition domain and T-cell signaling domain. T cells are genetically modified to express CARs, which are able to identify specifically target antigens on tumor cells. The FDA and EMA have approved CAR-T therapy targeting CD19 for the treatment of relapsed or refractory acute lymphoblastic leukemia and diffuse large B cell lymphoma. This documented success of CAR-T therapy against acute leukemia or lymphoma has encouraged the development of CAR-T treatment for MM, since several phase I, II, and III clinical studies of BCMA-targeting CAR-T cell therapy in heavily pretreated RRMM patients showed a high and deep response; although no plateau in PFS curves has been seen until now [28,29,30,31,32]. To date, CAR-T cell therapy for MM patients is experimental, and several clinical trials of CARs targeting alternative plasma cell antigens are ongoing.

This is a very exciting era for multiple myeloma research, and improved understanding of MM biology has led to achievements in the development of several different compounds. In the last decade, we have seen the transition from traditional chemotherapy towards more specific target drugs that are directed against precise clonal plasma cell surfaces’ antigens. This is the most important change in the recent history of hematologic disease therapy, especially for MM treatment. The available data suggest that T-cell immunity is crucial in controlling MM proliferation and survival, therefore a better understanding in MM pathophysiology and the crucial role of the immune system will be necessary. The hope is that immunotherapy, either passive or active, will modify MM outcome and will make MM a curable disease.

In this Special Issue, we highlight the current challenge and the future opportunities for the treatment for NDMM and RRMM, and we will try to provide an overview of currently approved immunotherapy drugs, as well as emerging immunotherapies in different phases of clinical research in MM. 
